# Understanding the Heavy Metal Pollution Pattern in Sediments of a Typical Small- and Medium-Sized Reservoir in China

**DOI:** 10.3390/ijerph20010708

**Published:** 2022-12-30

**Authors:** Qibei Bao, Cheng Liu, Kurt Friese, Tallent Dadi, Juhua Yu, Chengxin Fan, Qiushi Shen

**Affiliations:** 1 Ningbo College of Health Sciences, Ningbo 315100, China; 2 State Key Laboratory of Lake Science and Environment, Nanjing Institute of Geography and Limnology, Chinese Academy of Sciences, Nanjing 210008, China; 3 UFZ-Helmholtz Centre for Environmental Research, Department of Lake Research, 39114 Magdeburg, Germany; 4 Fujian Academy of Agricultural Sciences, Institute of Soil and Fertilizer, Fuzhou 350013, China; 5 Sino-Africa Joint Research Center, Chinese Academy of Sciences, Wuhan 430070, China

**Keywords:** heavy metal, sediment, source apportion, positive matrix factorization receptor model, pollution pattern, Tongjiqiao Reservoir

## Abstract

Heavy metal pollution in sediments is a common environmental issue in small- and medium-sized reservoirs not only in China but also worldwide; however, few interpretations of the pollution pattern exist. Based on the analyses of accumulation characteristics, ecological risks, and source apportionments of eight heavy metals (As, Cd, Cr, Cu, Hg, Pb, Ni, and Zn) in sediments, we derived a paradigm to describe the pollution pattern of heavy metals in sediments of a typical small- and medium-sized Tongjiqiao Reservoir. The results showed high levels of Cd, Hg, and As pollutants in the surface and upper sediment layers of the pre-dam area. Additionally, As, Cd, Hg, and Pb pollutants peaked in the middle layers of the inflow area, indicating a high ecological risk in these areas. The positive matrix factorization results implied that industrial, agricultural, and transportation activities were the main sources of heavy metals. The heavy metal pollution pattern exhibited three distinct stages: low contamination, rapid pollution, and pollution control. This pattern explains the heavy metal pollution process in the sediments and will provide scientific guidance for realizing the green and sustainable operation and development of the reservoir.

## 1. Introduction

Toxic and trace heavy metals from natural and anthropogenic inputs may pose a considerable threat to aquatic environments. These metals are detrimental to both aquatic organisms and human health in many ways because of their bioaccumulation, acute and chronic toxicity, and persistence [1,2,3,4]. Heavy metals enter surface water bodies via surface runoff, atmospheric deposition, or direct discharge [5,6,7], and then settle through sedimentation [8,9,10]. The environmental damage and ecological risks associated with sediments polluted with toxic and hazardous heavy metals (e.g., As, Cd, Cr, Cu, Hg, Ni, Pb, and Zn) have led to global concerns on the fate of various inland water bodies, including lakes, rivers, and reservoirs [9,10,11,12,13,14,15,16,17,18,19,20]. Reservoirs are more strongly influenced by and closely related to human activities than natural water bodies. Therefore, heavy metal pollution in reservoirs has attracted significant attention from scholars in the last several decades; however, investigations into the heavy metal pollution of sediments are more commonly found in China than anywhere else [8,17,21,22,23,24,25,26,27,28]. Studies from Danjiangkou Reservoir, the largest water source reservoir in China, with an area of 1050 km^2^ and total volume of 29.05 billion m^3^, showed that its surface sediments are polluted by Cd and Cr, with high ecological risks in some bays and tributaries [29]. The accumulation and pollution of heavy metals in sediments from other large reservoirs in China, such as the Three Gorges Reservoir of the Yangtze River, Liujiaxia and Xiaolangdi Reservoirs of the Yellow River, cascade reservoirs of the Lancang River, and Miyun Reservoir of Beijing, have also been reported [25,30,31,32,33,34,35,36,37]. Unfortunately, these reports mainly focused on heavy metal pollution and the correlated ecological risks in surface sediments; thus, the understanding of the accumulation process of heavy metals in reservoir sediments remains inadequate.

Compared with studies on large reservoirs, fewer studies on the heavy metal pollution in sediments of small- or medium-sized reservoirs have been reported both within and outside China [21,26,38,39]. Approximately 98,002 small- and medium-sized reservoirs (storage capacity range, 100,000–100 million m^3^), accounting for over 95% of the total number of reservoirs, exist in China [40]. Most of these small- and medium-sized reservoirs were built in the 1950–1970s and played an important role in providing a source of drinking water, irrigation, industrial activities, and aquaculture. Along with the extensive economic development spanning over 30 years since the 1980s, these reservoirs have been subject to pressure from various environmental issues, including heavy metal pollution in sediments [19,38,41,42]. The large reservoirs often have the dual nature of rivers and lakes, and their pollutant distribution and migration transformation processes are more complex. Compared with large reservoirs, small reservoirs are also mainly river-type reservoirs, but its lake-like properties are more obvious. Since the watersheds of the small- and medium-sized reservoirs are usually small, heavy metal contamination in the sediments of theses reservoirs often corresponds well to the socio-economic development and environmental protection efforts in the small region where the reservoir is located [43]. Heavy metal pollution in these reservoir sediments could have direct and intense environmental and ecological consequences. However, this issue has been given insufficient attention in previous decades. A well-known fact concerning heavy metals in reservoir sediment is that the release of heavy metals into overlying waters [44,45,46] can have adverse effects on aquatic organisms under certain conditions [8,9,47]. Previous studies confirmed that overloading sediments with heavy metals, even without release, can induce risks to the environmental health and ecological security of aquatic ecosystems [18,22,32]. However, most of these findings are based on investigations of heavy metals in surface sediments and lack an understanding of the contamination status of deep sediments. Few studies have discussed the potential sources or pollution processes of heavy metals in sediments using vertical distribution data in sediment cores from reservoirs [21,36,48]. Thus, some important questions remain unanswered: What drives heavy metal pollution in sediments of small- and medium-sized reservoirs? Is there a relatively universal pattern for this pollution process?

In this study, Tongjiqiao Reservoir was considered as a case study to investigate the spatial and vertical distribution characteristics of eight heavy metals (As, Cd, Cr, Cu, Hg, Pb, Ni, and Zn) in sediments. The pollution status and ecological risk of these heavy metals in the sediments were then evaluated using a contamination factor and potential ecological index methods [11,13,20,42]. Pollution sources of the heavy metals evaluated in different sediment layers were apportioned using the positive matrix factorization (PMF) receptor model, which was developed by the Environmental Protection Agency of the United States of America (US–EPA) and is proved to be an effective method for source apportion of heavy metals and other pollutants in sediments and soils [19,49,50]. Based on these analyses, we aim to obtain a clear understanding of the accumulation features of these heavy metals in the reservoir sediments and determine a relatively universal pattern to describe the pollution process of heavy metals in the sediments of this typical small- and medium-sized reservoir. The results of this study will provide new knowledge for understanding the pollution mechanism of heavy metals in sediments and developing suitable treatment methods for polluted sediments in reservoirs.

## 2. Materials and Methods

### 2.1. Research Area

**Figure 1:**
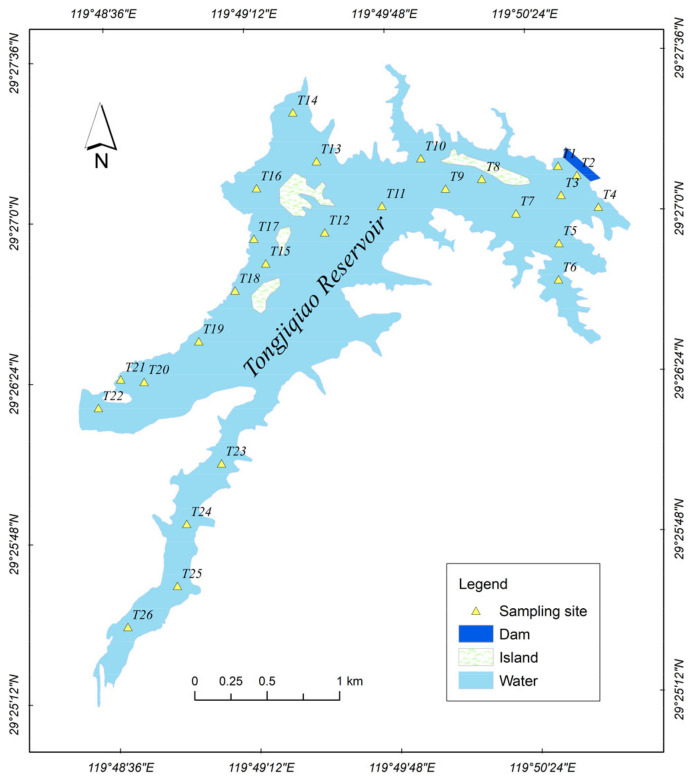
Sampling sites.

The study area, Tongjiqiao Reservoir, is located in Pujiang County, Jinhua City, Zhejiang Province, which is an economically developed area on the eastern coast of China (29.420°–29.459° N, 119.802°–119.847° E), as shown in Figure 1. The Tongjiqiao Reservoir is a typical medium-sized reservoir in a township, with a total volume of 80.76 million m^3^. The surface area of the reservoir is approximately 4.5 km^2^, and the average and maximum depths of the reservoir are 27 and 48 m, respectively. The reservoir lies in a subtropical monsoon climate with an annual precipitation of 1412.2 mm and average annual temperature of 16.6 °C. The reservoir was built in 1958 and was one of the first “eight reservoirs” approved for construction in Zhejiang Province. The reservoir is located in the upper reaches of the Puyang River, a tributary of the Qiantang River, and the dam site is 4.0 km from the center of Pujiang County. The downstream irrigation area of the reservoir is mainly a grain-producing area with agricultural development projects, including 57.34 km^2^ of good arable land, accounting for 45% of the county’s irrigated area. Approximately 219,000 people, accounting for 57% of the county’s population, are involved in agricultural irrigation activities and ecological water.

The Tongjiqiao Reservoir is the backbone storage reservoir of the main watercourse of the Puyang River. The reservoir receives flood water and sediments from numerous upstream tributaries, causing sediment accumulation in the reservoir area and raising the water level. No siltation cleaning has been conducted since the construction of the reservoir, and long-term accumulation has led to a rapid thickening of the sediment. The maximum contents of total phosphorus and nitrogen in the sediments of the Tongjiqiao Reservoir are 6391 and 6915 mg/kg, respectively. The maximum content of organic matter is 20%, with the average content exceeding 8%. Thus, the reservoir is considered to be in a moderate nutrient state in terms of water quality.

### 2.2. Sampling Sites

Considering the shoreline morphology of the Tongjiqiao Reservoir, water pollution degree, water area, spillway, tributaries, and dam influencing factors, 26 sediment sampling sites were established throughout the reservoir, as shown in Figure 1. The sampling sites were recorded as T1–T26. Geographically, the Tongjiqiao Reservoir is divided into the pre-dam region (notation D, sites T1–T3), central and eastern regions (notation CE, sites T4–T12), north region (notation N, sites T13–T18), west region (notation W, sites T19–T22), and southwest bay region (notation SW, sites T23–T26). The southwest bay region is the inflow area of the reservoir.

### 2.3. Sample Collection and Storage

A sediment column was collected from each sampling site using a gravity bottom sampler (Ø 8.4 cm × L 50 cm). The sediment column was divided into 1 cm intervals for the upper 10 cm and at 2 cm intervals thereafter. The sediment sub-samples were sealed in polyethylene bags by number and stored below 25 °C for transport to the laboratory. The 0–2 cm layer was considered the surface sediment, denoted as S. The 2–5 cm layer was considered the upper sediment, denoted as U. The 5–10 cm layer was considered the middle sediment, denoted as M. The 10 cm layer below the middle sediment was considered the lower sediment, denoted as L. In total, 26 sediment column samples and 241 sub-samples were collected for this sampling. The air-dried samples were homogenized and pulverized using an agate mortar and pestle and screened through a 200-mesh nylon sieve. All samples were stored in containers at 25 °C.

### 2.4. Chemical Analysis and Quality Control

The samples were analyzed according to the relevant standards issued by the United States Environmental Protection Agency (US–EPA). After microwave-assisted digestion, the levels of As, Cd, Cr, Cu, Ni, Pb, and Zn were determined using inductively coupled plasma mass spectrometry (Agilent 7700X, Agilent Co., Santa Clara, CA, USA) according to the US–EPA Method 6020a (Revision 1, February 2007). Hg was determined using a Hydra II C fully automated Hg meter (Teledyne Leeman Labs, Mason, OH, UAS) according to US–EPA Method 7473 (1998). The limits of detection for As, Cd, Cr, Cu, Hg, Ni, Pb, and Zn were 0.1, 0.01, 0.1, 0.02, 0.0006, 0.05, 0.02, and 0.2 mg/kg, respectively.

The average recovery of the certified reference material ranged from 92% to 105%. No fewer than 10% of the samples were tested in triplicate to ensure measurement precision, and the results showed that the relative deviations were less than 5%.

### 2.5. Sediment Contamination and Ecological Risk Assessment

#### 2.5.1. Contamination Factor ($C_{f}^{i}$)

The contamination factor ($C_{f}^{i}$) is usually used to assess the contamination level of potentially toxic elements in sediments. $C_{f}^{i}$ is calculated as follows:

(1) $$C_{f}^{i}=C_{s}^{i}/C_{n}^{i}$$

where $C_{s}^{i}$ is the average concentration (mg/kg) of heavy metal *i* in the sediments and $C_{n}^{i}$ is the geochemical background concentration(mg/kg) of heavy metal *i*. The $C_{f}^{i}$ index can be divided into four classes: low contamination ($C_{f}^{i}$ < 1), moderate contamination (1 ≤ $C_{f}^{i}$ < 3), considerable contamination (3 ≤ $C_{f}^{i}$ < 6), and very high contamination ($C_{f}^{i}$ ≥ 6).

#### 2.5.2. Potential Ecological Risk

The risk index (RI) was introduced by Hakanson to comprehensively assess the ecological risk of heavy metals in sediments **[51,52]**. This method considers the variation in background values, as well as environmental sensitivity and heavy metal toxicity. RI is calculated using the following equation:

(2) $$E_{r}^{i}=T_{r}^{i}\times C_{f}^{i}$$

(3) $$RI=\sum_{i=1}^{n}E_{r}^{i}$$

where $C_{f}^{i}$ is the contamination factor of heavy metal *i*, T_r_^i^ is the toxicity coefficient of a certain heavy metal, and $E_{r}^{i}$ is the potential ecological assessment coefficient for a certain metal *i*. Based on previous research [53], the toxicity coefficients of As, Cd, Cr, Cu, Hg, Pb, Ni, and Zn used in the analysis were 10, 30, 2, 5, 40, 5, 5, and 1, respectively. $E_{r}^{i}$ can describe five potential ecological risk levels, namely, low ($E_{r}^{i}$ < 40), moderate (40 ≤ $E_{r}^{i}$ < 80), considerable (80 ≤$\;E_{r}^{i}$ < 160), high (160 ≤ $E_{r}^{i}$ < 320), and very high ($E_{r}^{i}$ ≥ 320). The categories for RI are low (RI < 110), moderate (110 ≤ RI < 220), high (220 ≤ RI < 440), and very high (RI ≥ 440).

### 2.6. Source Apportionment

The pollutant sources were identified and apportioned using PMF Receptor Model 5.0, which was released by the US–EPA. The sample concentration data matrix of the input model was divided into two matrices, namely, factor contribution (G) and factor component spectrum (F), and the number of factors and factor contributions were identified using a multilinear multiple iteration (ME) algorithm. Data below the method detection limit (MDL) were set to MDL/2, with an uncertainty of 5/6 of the corresponding MDL. Data less than the MDL were used with uncertainty values equal to the CSN error with the addition of 1/3 of the MDL. The PMF model calculated the contribution of each factor by the percentage of the variance of the contribution matrix of each factor. The detailed PMF computation procedure was described by Norris et al. [50].

### 2.7. Data Analysis

All statistical analyses were performed using SPSS 19.0 (IBM® SPSS® Statistics, Armonk, NY, USA). All figures were plotted using OriginLab2021b (OriginLab Corporation, Northampton, MA, USA) and ArcGIS10.7 software (Environmental Systems Research Institute, Inc., Redlands, CA, USA).

## 3. Results and Discussion

### 3.1. Spatial Distribution of Heavy Metals in Surface Sediments

Descriptive information for As, Cd, Cr, Cu, Hg, Ni, Pb, and Zn in the sediments of the study area, as well as the geogenic background values in the local soil, are listed in Table 1. The average concentration of heavy metals in the sediments decreased in the order Zn (136 mg/kg) > Pb (67.0 mg/kg) > Cr (53.2 mg/kg) > Cu (30.7 mg/kg) > As (22.4 mg/kg) > Ni (18.4 mg/kg) > Cd (0.95 mg/kg) > Hg (0.41 mg/kg). All heavy metal concentrations, except those of Cr and Ni, exceeded the corresponding local background levels. In comparison with published data from other reservoirs and lakes in previous studies (Table 1), the As and Hg contents of the sediments of the study area were significantly higher, ranging from 5.95 to 16.8 mg/kg for As and 0.03 to 0.13 mg/kg for Hg [10,12,14,16,34,43]. These results indicate the strong influence of anthropogenic activities on trace element pollution in the Tongjiqiao Reservoir.

**Figure 2:**
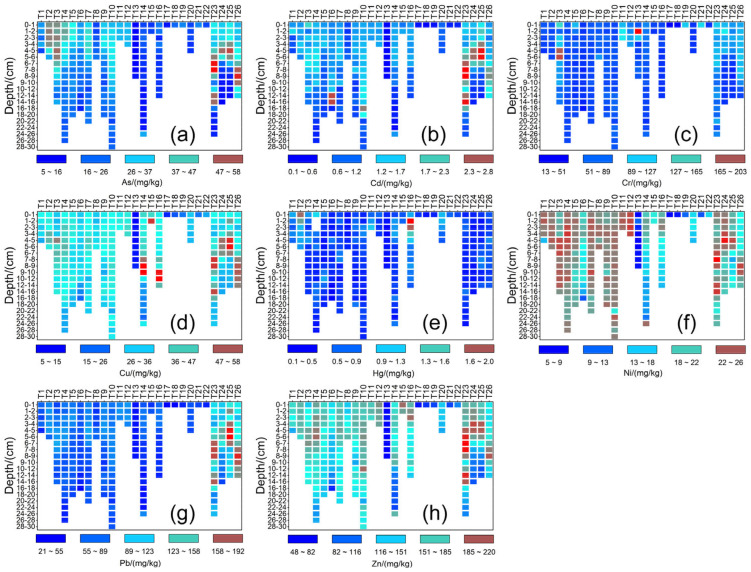
Distribution of heavy metals in sediments of Tongjiqiao Reservoir. (**a**–**h**) represent different heavy metals of As, Cd, Cr, Cu, Hg, Ni, Pb, and Zn, respectively.

The coefficient of variation (CV) for Hg (59.96%), Cd (56.96%), and As (50.30%) in the whole reservoir demonstrated the significant spatial heterogeneity of these metal concentrations. The CV values of the other trace metals were in the range of 21.90–48.37%. The spatial distribution of heavy metals in the sediment (Figure 2) indicated that the pollutants tended to be concentrated in the D and SW areas of the reservoir. The concentrations of heavy metals in the D and SW areas were 19–105% and 21–128% higher on average, respectively, than those in the CE, N, and W regions.

Heavy metal accumulation in the D region may be the result of incoming pollutants that migrate with the flow field and hydrodynamic conditions and then settle in front of the dam. The accumulation of pollutants in the SW region may be related to the development of processing and manufacturing industries in the surrounding villages and towns. Additionally, the concentrations of As (22.18 mg/kg), Cd (0.90 mg/kg), and Cr (46.69 mg/kg) in the CE region were second only to those in the D and SW regions, indicating that these pollutants in the CE region should also be of concern. Hg concentrations were highest in sediments at T16 in the N region, which suggests the influence of a particular source.

### 3.2. Vertical Distribution of Heavy Metals in Sediments

Figure 2 shows the vertical distribution of heavy metals in each region of the reservoir. The average As contents in the 0–5 cm sediment layer of the D, CE, N, W, and SW regions were 34.2 ± 11.2, 27.3 ± 6.8, 12.4 ± 4.7, 14.3 ± 3.5, and 33.4 ± 7.2 mg/kg, respectively. The As concentration in each column in the reservoir tended to increase with increasing depth, reached a maximum value, and then decreased thereafter. Notably, the As content of T23 in the SW region gradually increased with increasing depth in the 0–10 cm layer, peaked at 57.7 mg/kg at 8–9 cm, and then decreased with further increases in depth. However, high concentrations of the metal (45.7 and 47.1 mg/kg) were observed in the 14–18 cm layer, followed by a gradual decrease. This result indicates that a point source of As input may be present around the sampling site. The As content in the lower sediment layer of all regions decreased significantly at depths below 10 cm. Indeed, the As content at this depth was significantly lower than the background content of As (9.7 mg/kg) in the soil of the Jinhua–Quzhou region of Zhejiang.

The Cd concentration of all sediment column samples was relatively high in the surface, upper, and middle layers (0–10 cm) and then gradually decreased. The peak Cd concentration of most samples occurred in the 0–5 cm layer, except in T6 and T10 in the CE region and T23 in the SW region. In the CE region, peak Cd concentrations of 2.40 and 2.11 mg/kg were found in the 14–18 cm layer; in the SW region, the peak Cd concentration appeared in the 9–10 cm layer. For column samples with sediments at depths below 20 cm, the Cd content was near or lower than the background soil Cd content (0.274 mg/kg) in the Jinhua–Quzhou region. The surface, upper, and middle sediment layers were formed at a relatively late period, during which the water quality and reservoir sediment were significantly affected by industrial and agricultural developments. Anthropogenic inputs and discharges in this later period were the main reasons behind the increase in Cd accumulation in these layers. Sokołowska and Kulbat [26] reported that Cd was the most significantly accumulated heavy metal in the sediments of drinking water reservoirs in Poland. After studying five small- and medium-sized reservoirs in Northeast China, Zhu et al. [55] similarly concluded that Cd was the main heavy metal accumulated in the sediments at various depths.

Two types of vertical distributions of Cr were observed in the sediment column samples. In the first type, the Cr content was relatively high in the surface and upper layers (0–5 cm) but markedly decreased at depths beyond 5 cm. This type of distribution was mainly located in the D and SW regions (T3, T5, T13, and T23–T26). In the second type, the Cr content was generally lower than the background soil Cr content (60.6 mg/kg) in the Jinhua–Quzhou region and remained stable with increasing depth.

The Hg content of all sediment column samples was higher than the background soil Hg content (0.15 mg/kg) in the Jinhua–Quzhou region. In terms of vertical distribution, the Hg contents in the 0–5 cm layer of the D and N regions (average concentrations of 0.7 and 1.3 mg/kg, respectively) were higher than that in the same layer of the other regions (average concentration of 0.5 mg/kg). After peaking at a certain value, the Hg content gradually decreased with increasing depth. However, the Hg content remained higher than the background Hg content in the local soils. The presence of Hg in sediments collected at various depths indicates a significant level of Hg pollutant accumulation.

The Cu, Ni, Pb, and Zn contents in the sediment column samples presented two types of vertical distributions. The contents of these heavy metals gradually increased in the surface and upper layers (0–5 cm) of the SW region and then significantly decreased with increasing depth beyond 5 cm. This finding may be related to increased Cu, Ni, Pb, and Zn emissions around the SW region over a certain period after reservoir formation. In the other regions, the Cu, Ni, Pb, and Zn contents in the sediments remained stable with increasing depth and were lower than the corresponding background values (Cu, 24.8 mg/kg; Ni, 18.5 mg/kg; Pb, 42.4 mg/kg; Zn, 95 mg/kg) in the Jinhua–Quzhou region.

### 3.3. Ecological Risks of Heavy Metals

**Figure 3:**
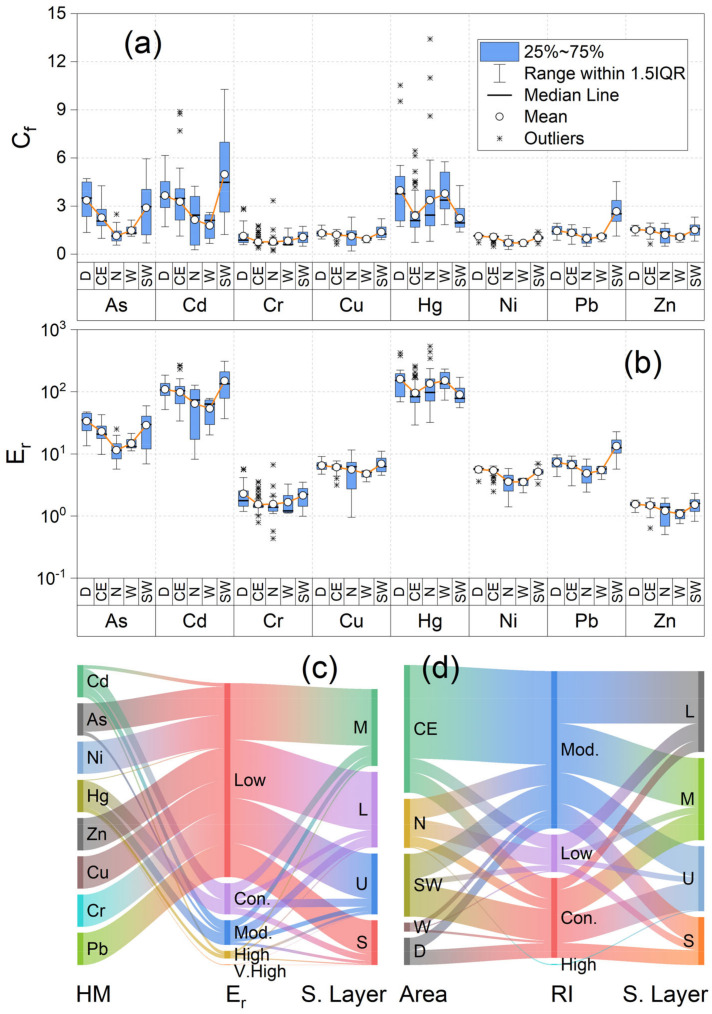
Contamination level and potential ecological risk of heavy metals in sediments of the Tongjiqiao Reservoir: (**a**) boxplot of C_f_ for heavy metals, (**b**) boxplot of E_r_ for heavy metals, (**c**) Sankey plot of E_r_ for heavy metals, and (**d**) Sankey plot of RI for heavy metals. In (**a**,**b**), D, CE, N, W, and SW represent areas next to the main dam, central and eastern, northern, western, and southwestern regions of the reservoir, respectively. In (**c**,**d**), Low, Mod., Con., High, and V. High signify that E_r_ or RI are at a low, moderate, concentrated, high, or very high level/risk, respectively. S, U, M, and L represent the surface, upper layer, middle layer, and low layer sediments, respectively. S. Layer represents the sediment layer.

The single contamination factor index of the sampling sites at different depths is an important measure reflecting the degree of sediment pollution by a particular pollutant; its value for a heavy metal can be used to classify the pollution level by that pollutant. The single contamination factor indices of the eight heavy metal pollutants in the sediments of the different reservoir regions at various depths are shown in Figure 3a,c. The pollution indices of As, Cd, Cu, Hg, Pb, and Zn in all sampling sites were greater than 1, indicating the accumulation and pollution of these heavy metals in the reservoir area. The average value of the pollution indices of these six heavy metals presented a decreasing trend with increasing depth. In terms of spatial distribution, the accumulated pollution of the heavy metals was most concentrated in the D and SW regions. The survey results of the samples collected from most of the regions indicated no Cr or Ni pollutants, except in the D and SW regions, where the Cr and Ni pollution indices exceeded 1 in some sampling sites.

The biotoxicity risk assessment results of each heavy metal in the sediments at various depths determined using the potential ecological risk index method are shown in Figure 3b,d. According to the E_r_ values of the various heavy metals in the sediments in various layers, As, Cd, and Hg were the main sources of ecological risk; among these metals, the overall potential ecological risk of Cd and Hg were greater than that of As, consistent with the results presented by Ma [14] and Kuang [42] after studying small- and medium-sized reservoirs in China. From the spatial distribution perspective, As in the 0–5 cm layer of the D region posed a considerable potential ecological risk, whereas that in the remaining layers only posed a low potential ecological risk. Cd posed a high potential ecological risk in 83% of the sampling sites at various depths and a considerable potential ecological risk in the remaining 17%. The potential ecological risks of Hg were very high, high, and considerable in 46%, 21%, and 33% of the sampling sites, respectively.

The potential ecological risks of Cd and Hg in the surface, upper, and middle sediment layers were generally high. Specifically, a high potential ecological risk of Cd and Hg was observed in sediments of the 0–5 cm layer of the CE region and 0–10 cm layer of the N region. The potential ecological risks of Cd and Hg were especially high at T4, T5, T6, T7, and T10 in the CE region, even affecting sediments 20 cm below the water–soil interface. In the W region, only the 0–5 cm layer showed a strong potential ecological risk of Hg. The potential ecological risks of Cd and Hg in the surface and upper sediment layers of the SW region were generally high; specifically, in the 0–5 and 0–10 cm layers, these risks reached high/very high and high levels, respectively. A considerable potential ecological risk of As was also observed in the 0–10 cm layer. The potential ecological risk of Cd in this region was greater than those of As and Hg. Cd was found in the middle sediment layers of T23, T24, and T26, all showing very high Cd levels.

### 3.4. Potential Ecological Risks of Sediments

**Figure 4:**
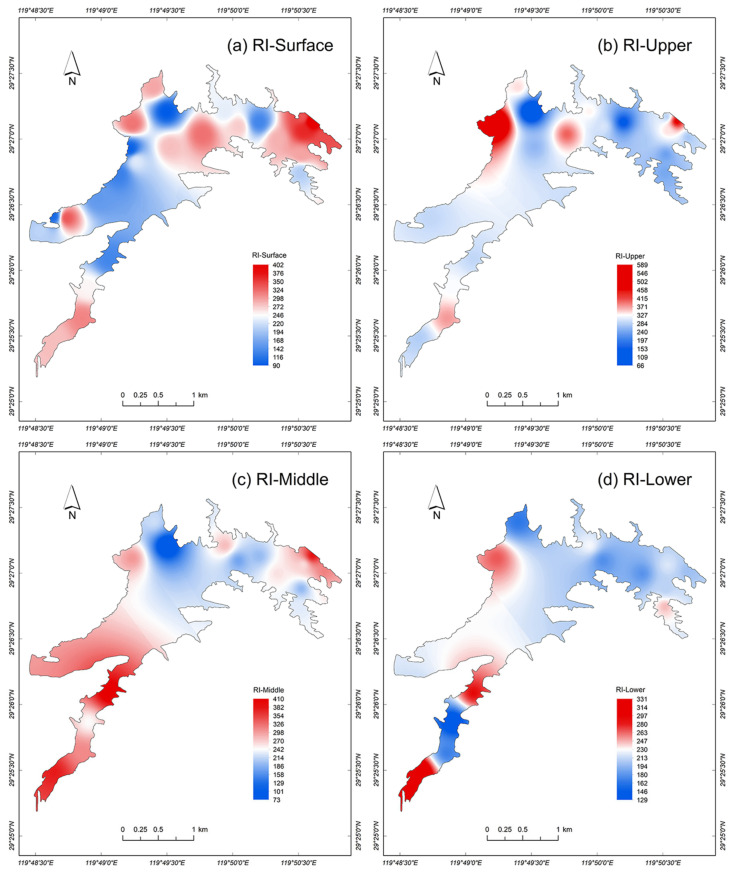
Potential ecological risk of sediments from heavy metals of the Tongjiqiao Reservoir: Distribution of the risk index (RI) of sediments in the (**a**) surface, (**b**) upper, (**c**) middle, and (**d**) lower sediment layers.

The comprehensive potential ecological risk assessment results for heavy metals in the sediments of the Tongjiqiao Reservoir indicate a certain degree of potential risk at various depths (average RI, 258), with the spatial distribution of sediment risk areas in the various layers differing between the SW and D regions. A remarkable characteristic of the distribution of high potential ecological risk is the gradual shift from the SW to the D region with decreasing sediment depth (i.e., over time). Specifically, areas with potential ecological risk of heavy metals in the surface sediment layer were concentrated in the D and CE regions (Figure 4a) (average RI, 326), whereas those in the upper sediment layer were mostly distributed in the N region (Figure 4b). In the middle sediment layer, the main potential risks were distributed in the SW and W regions (Figure 4c). For sediments deeper than 10 cm, the main potential risks were in some parts of the SW region (Figure 4d; average RI, 298). These spatial variations are mainly related to the source routes of heavy metals to the reservoir and their input, transport, and deposition processes.

Among the sampling sites with a high risk of heavy metals, 40% were located in the SW region. The proportions of sampling sites located in the CE, D, N, and W regions were 26%, 19%, 13%, and 3%, respectively. In terms of vertical distribution, the high-risk sites in the various regions were concentrated in the surface, upper, and middle sediment layers (0–10 cm). Only 16% of the sampling sites with high-risk points were distributed in the lower sediment layer (below 10 cm). The spatial distribution of high-risk areas was highest in the SW region (8.6%), followed by the CE (5.7%) and N (1.4%) regions.

The source composition of heavy metals in sediments indicated that Hg and Cd were the main sources of ecological risk. The combined contribution rate of these two heavy metals in the sediments at a depth of 0–10 cm was 64.2–94.5%. Previous studies indicated that most lake-reservoir areas in China are surrounded by irrigated cultivated land; thus, Cd was a major risk factor for lake-reservoir water quality and heavy metals in sediments [10,14], which could be related to factors such as the cultivation and fertilization methods employed. Although the Hg and Cd concentrations obtained were relatively lower compared with those of the other heavy metals tested, they represent stronger ecological risks because of their greater toxicity and higher toxicity coefficients, which significantly contribute to their overall potential risk. In the D, N, and W regions, the ecological risk contribution rate of Hg in the surface, upper, and middle sediment layers was relatively high. In the CE and SW regions, the order of the contribution rates of potential risk in the 0–10 cm layer was Cd > Hg > As, whereas that in the lower sediment layer (below 10 cm) was Hg > Cd > As. For the CE and SW regions, differences in the inputs of heavy metal pollution were more apparent; Hg inputs were mainly observed in the early stages of pollution, whereas Cd inputs dominated in the later stages.

### 3.5. Source Apportionment of Heavy Metals

**Figure 5:**
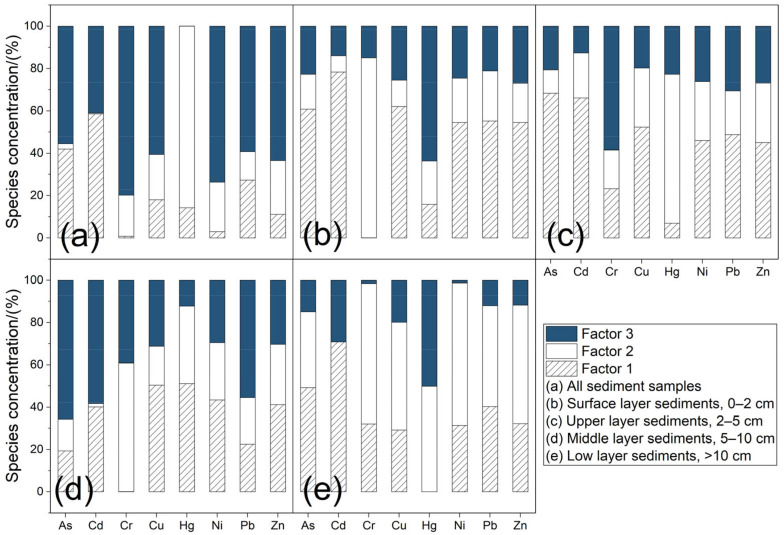
Source apportionment results for different heavy metals using the PMF model: Analysis results of the (**a**) overall contribution, (**b**) surface sediment layer, (**c**) upper sediment layer, (**d**) middle sediment layer, and (**e**) sediment layer below 10 cm. Factor 1: agricultural activities; Factor 2: traffic emissions and fuel combustion; Factor 3: industrial activities.

The source composition spectrum of each heavy metal in the sediments, which was obtained through PMF analysis, is shown in Figure 5. Based on the results, three factors were selected to explain the categories of pollution sources. In terms of the overall contribution of the eight heavy metals in all sediment samples (Figure 5a), the relative contribution rate of Factor 1 to Cd was the highest at 58.6%, and that of Factor 2 to Hg was the highest at 85.8%. Factor 3 was the main contributor to As, Cr, Cu, Ni, Pb, and Zn in the sediments.

After a comprehensive analysis, Factor 1 was determined to be agricultural activities, such as fertilization of modern cultivated and forested land, which led to Cd inputs. Concomitantly, the excessive use of agricultural pesticides, chemical fertilizers, and organic fertilizers also led to Cu, Zn, and Pb accumulation. Factor 2 was determined to be vehicular emissions during transport or exhaust emissions from industrial fuel combustion, which led to Hg input. This heavy metal entered the reservoir waterbody through atmospheric dry–wet deposition and surface water runoff, eventually accumulating in the sediments. Factor 3 was determined to be industrial activities, which led to the inputs of As, Cr, Cu, Ni, Pb, and Zn.

The results of the analysis of heavy metal sources in the surface and upper sediment layers are shown in Figure 5b,c. Factor 1 was the greatest contributor of heavy metals in the surface and upper sediment layers at 47.6% and 44.6%, respectively. The main source of heavy metals in these two layers was agricultural activities such as the fertilization of modern cultivated and forested land, which resulted in Cd inputs. The excessive use of pesticides, chemical fertilizers, and organic fertilizers for cultivated land also resulted in Cu, Zn, and Pb accumulation.

The results of the PMF analysis for the middle sediment layer (5–10 cm layer) are shown in Figure 5d. Factor 3 was the greatest contributor (40.3%) to the accumulation of heavy metals, followed by Factor 1 (contribution rate, 33.5%) and Factor 2. Thus, industrial activities were the main source of As, Cr, Cu, Ni, Pb, and Zn inputs in the middle sediment layer. The artificial crystal industry (the local pillar industry) contributed to As inputs, and the padlock production industry contributed to the inputs of Cu and Zn. The average contribution rate of Factor 2 reached 46.7% for the sediment layer below 10 cm, followed by Factors 1 and 3 with contribution rates of 35.6% and 17.7%, respectively. The reservoir was built some time ago when many factories had been established in the surrounding area, including transportation routes passing through the reservoir area. Hg emissions in exhaust emissions from the early period—before the use of improved gasoline or fuel—are expected to be relatively high. Thus, the source of heavy metals in the sediment layer below 10 cm (Figure 5e) was mainly Hg input, either from exhaust emissions from automobile transportation or the combustion of industrial fuels.

### 3.6. Heavy Metal Pollution Patterns and Management Suggestions

As of 2011, China has 98,002 reservoirs, of which more than 95% are small- and medium-sized reservoirs with storage capacities ranging from 100,000 to 100 million m^3^ [40]. Tongjiqiao Reservoir, which is built in the upper reaches of the Puyang River in a mountainous area, is typical of such reservoirs. Similar to most other small- and medium-sized reservoirs built in China between the 1950s and 1970s, its main functions include cultivated land irrigation and hydroelectric power generation, as well as water supply for industrial use and aquaculture. The area surrounding the small watershed of the reservoir is dominated by cultivated and forested land, and a sizable scale of industrial enterprises can be found in the upper reaches of the Puyang River, including papermaking, printing and dyeing, electroplating, and livestock and poultry breeding. These agricultural and industrial activities flourished along with China’s reform and opening up policy after 1979. At this time, Pujiang County, where the Tongjiqiao Reservoir is located, gradually developed into a center for crystal processing and padlock production, both of which accounted for over 70% of the total sales in the Chinese market. Previous studies reported that the main heavy metal elements in the crystal waste from Pujiang were As, Cr, Ni, and Zn, and that the As content reached 388–584 mg/kg [42].

**Figure 6:**
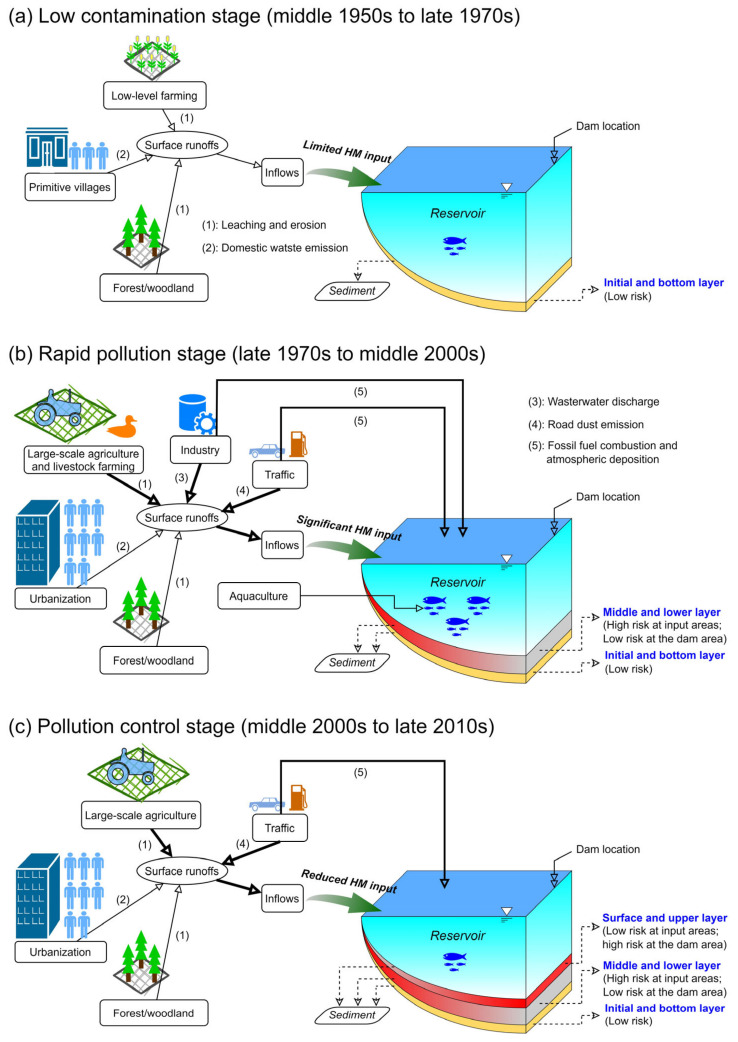
General pattern of heavy metal pollution processes in sediments of typical small- and medium-sized reservoirs: (**a**) low contamination stage, (**b**) rapid pollution stage, and (**c**) pollution control stage.

The source apportions of heavy metals in our study clearly demonstrated that the main sources of heavy metal pollutants in sediments of the reservoir are emissions and inputs from industrial, agricultural, and other anthropogenic production activities in the catchment area, and differed for each sediment layer. Therefore, a particular pollution pattern comprising three stages of heavy metal accumulation in the sediments of the reservoir can be derived (Figure 6). The first stage is the low contamination stage (from middle 1950s to late 1970s), which spans the time from reservoir completion until the start of China’s reform and opening up, during which agricultural production was primitive and inefficient, prior to the emergence of industrial production. The level of heavy metal pollution in the sediments was very low, with the main pollution sources being inputs from the geogenic background of the basin (Figure 6a). The second stage is the rapid pollution stage (from late 1970s to middle 2000s), which spans the time after the reform and opening up, during which rapid developments in small- and medium-sized enterprises and large-scale agricultural production in villages and towns occurred. Heavy metal pollution in the sediments increased sharply. The main sources of pollutants were industrial and agricultural production, accompanied by inputs from traffic pollution (Figure 6b). The third stage is the pollution control stage (from middle 2000s to late 2010s), which began at the start of the 21st century with the growing scale and intensity of environmental pollution control; in this stage, the discharge of pollutants into the basin was limited, thereby significantly reducing the degree of heavy metal pollution in the sediments (Figure 6c). The input of pollutants from anthropogenic activities—especially industrial inputs—declined sharply, leading to concomitant reductions in the degree of heavy metal pollution in the sediments. However, non-point source pollution caused by agriculture and economic forest planting remained severe.

Despite the alleviation in heavy metal inputs, obvious pollutant transfer trends between the various reservoir regions can be observed. Heavy metal pollution in sediments was characteristically transported from the reservoir inflow area (SW region in this study) to the pre-dam region (D region in this study). Consequently, the potential ecological risks of heavy metal pollution in the surface and upper sediment layers in the D region and its surrounding areas continuously increased. Moreover, the sediments in this region exhibited extensive deposition and pollution from nitrogen and phosphorus nutrients [36]. This type of complex sediment pollution and siltation caused obvious harm to the water ecology and safe operation of the reservoir.

The Tongjiqiao reservoir continues to function for cultivated land irrigation, flood control, power generation, and even drinking water supply; therefore, sediment management must be considered in efforts to comprehensively manage and upgrade the water environment. Furthermore, when improving and ensuring the water environment and ecological security of the reservoir, several principal tasks should be performed in addition to routine monitoring and maintenance. First, sediments that are already polluted, especially in problematic areas such as the D region, should be dredged. The harmful ecological risk of sediments would be reduced after removing endogenous pollution. Second, industrial pollution sources in the reservoir catchment area must be blocked and monitored to prevent pollutants from entering the reservoir and to reduce the input of toxic and harmful heavy metals from the largest sources. Third, pollution control should be implemented for agriculture and economic forest planting. The implementation of ecological agriculture should be strongly promoted to elevate the overall level of local ecological agriculture development, thereby effectively controlling non-point source agricultural pollution. Finally, the management of mobile pollution sources should be strengthened. The collection and disposal of garbage and sewage on road surfaces in the protected area must also be conducted in a timely manner to ensure pollutants do not enter the waterbody.

## 4. Conclusions

Pollutants of As, Cd, Cu, Hg, Pb, and Zn were widely found in both the surface and lower sediment layers of the Tongjiqiao Reservoir, a typical small- to medium-sized reservoir. The pre-dam and inflowing areas of the reservoir were identified as two hot spots for the accumulation of heavy metal pollutants. The high potential ecological risk of heavy metals in the sediments was mainly caused by Cd, Hg, and As in the entire reservoir. In addition, clear chronological changes in these risks were verified. The heavy metal contents and potential ecological risk to sediments in the inflowing area of the reservoir significantly increased during the rapid industrialization and urbanization periods and then dramatically decreased after the implementation of pollution control in the mid-2000s. However, the high risk was notably transferred to the pre-dam area. The results of the PMF source analysis indicated that, during the period of rapid industrialization, As, Cr, Cu, Ni, Pb, and Zn in the sediments of the middle layer were mainly produced by industrial activities. The heavy metals in the sediments below 10 cm were from exhaust emissions of automobile transportation or other fossil fuel combustion. After the implementation of pollution control, the heavy metals were mainly from agricultural activities.

A paradigm was summarized to describe the three-stage pattern of heavy metal pollution in sediments of the typical small- to medium-sized Tongjiqiao Reservoir. The low-pollution stage lasted from reservoir construction to the pre-industrial period and was dominated by heavy metal inputs from the geogenic background of the basin. The degree of pollution and potential ecological risk of the sediments were low at this stage. The accumulation of heavy metals in the inflowing area of the reservoir became apparent during the high-pollution stage, which is associated with rapid industrialization, agricultural modernization, and urbanization. The pollution control stage occurred after the implementation of industrial pollution control, during which heavy metal pollution in the sediments significantly decreased in the inflowing area; however, more heavy metals accumulated in the pre-dam area and correspondingly increased the ecological risk in this area.

Focusing on theoretical hot spots and pollution layers while implementing appropriate environmental management measures is important for controlling the heavy metal pollution in a reservoir. Treatment efforts such as sediment dredging should be based on investigations and evaluations of both the surficial and vertical distributions of the targeted pollutants. Furthermore, the environmental restoration of the reservoir should be strongly intertwined with the corresponding catchment management. The discharge of heavy metals from non-point sources of agriculture and economic forest planting within the watershed of reservoirs should be effectively controlled. Management of mobile pollution sources is indispensable and must be intensified to reduce heavy metal inputs from fossil fuel combustion.

## Figures and Tables

**Table 1:** Comparison of As, Cd, Cr, Cu, Hg, Ni, Pb, and Zn concentrations determined in this study, the geogenic background, and other reservoirs or drinking water lake sediments (mg/kg dry weight).

	As	Cd	Cr	Cu	Hg	Ni	Pb	Zn	Reference
^a^ Means	22.35	0.95	53.20	30.74	0.41	18.43	66.99	136.33	This study
^b^ Means (surface layer)	23.74	0.87	61.79	28.45	0.52	17.73	62.41	135.47	This study
Max	57.7	2.81	202	57.4	2.013	26.0	192.0	219.0	This study
Min	5.50	0.07	13.20	4.70	0.109	5.20	20.50	47.60	This study
SD	11.24	0.54	23.44	8.85	0.25	4.04	32.40	32.78	This study
^c^ CV (%)	50.30	56.96	44.05	28.78	59.96	21.90	48.37	24.05	This study
^d^ Background	9.7	0.274	60.6	24.8	0.15	18.5	42.4	95	[54]
^e^ National standard for soil quality	25	0.6	250	100	3.4	190	170	300	GB 15618-2018
Three Gorges Reservoir, China	10.08	0.33	140.3	97.72	0.05	35.17	114.1	177.0	[28]
Miyun Reservoir, China	/	0.3	77.9	38.0	/	/	29.3	106.8	[34]
Taihu Lake, China	11.46	0.78	223.30	43.02	0.10	54.63	45.48	153.09	[20]
Chaohu Lake, China	10.4	0.44	72.5	26.0	0.114	/	47.1	137.8	[46]
Shuanglong Reservoir, China	5.95–16.76	/	51.20–129.70	14.90–38.29	0.03–0.13	14.92–34.93	16.47–76.54	28.02–104.90	[43]
Dragon Lake, China	/	1.27	/	28.74	/	30.54	26.95	146.45	[14]
Longhu Lake, China	8–10.5	1.30–2.30	36–66.32	60–91.85	/	19–53.02	70–107.16	290–454.4	[16]
Hoedong Reservoir, Korea	/	1.6	28.7	57.6	/	17.2	60.5	247.8	[24]
EI Guajaro Reservoir, Colombia	/	/	/	/	0.06	/	9.8	125.1	[27]

^a^ Mean values of heavy metal content in all samples of all sampling sites. ^b^ Mean values of heavy metal content in surface samples of all sampling sites. ^c^ CV (%) = coefficient of variation. ^d^ The background values of trace elements were referenced from the Zhejiang Geological Survey Institute and Chinese Academy of Geological Sciences [54]. ^e^ National standards for soil quality were referenced from the National Standards of the People’s Republic of China (GB 15618-2018 Soil environmental quality—Risk control standard foil contamination of agricultural land).

## Data Availability

Not applicable.
